# Post-Translational Processing of Synaptophysin in the Rat Retina Is Disrupted by Diabetes

**DOI:** 10.1371/journal.pone.0044711

**Published:** 2012-09-06

**Authors:** Travis S. D’Cruz, Brittany N. Weibley, Scot R. Kimball, Alistair J. Barber

**Affiliations:** 1 Department of Ophthalmology, The Penn State Hershey Eye Center, Penn State Hershey College of Medicine, Milton S. Hershey Medical Center, Hershey, Pennsylvania, United States of America; 2 Cellular and Molecular Physiology, The Penn State Hershey Eye Center, Penn State Hershey College of Medicine, Milton S. Hershey Medical Center, Hershey, Pennsylvania, United States of America; Universidade Federal do Rio de Janeiro, Brazil

## Abstract

Synaptophysin, is an abundant presynaptic protein involved in synaptic vesicle recycling and neurotransmitter release. Previous work shows that its content is significantly reduced in the rat retina by streptozotocin (STZ)-diabetes. This study tested the hypothesis that STZ-diabetes alters synaptophysin protein turnover and glycosylation in the rat retina. Whole explant retinas from male Sprague-Dawley rats were used in this study. Rats were made diabetic by a single intraperitoneal STZ injection (65 mg/kg body weight in 10 mM sodium citrate, pH 4.5). mRNA translation was measured using a ^35^S-methionine labeling assay followed by synaptophysin immunoprecipitation and autoradiography. A pulse-chase study was used to determine the depletion of newly synthesized synaptophysin. Depletion of total synaptophysin was determined after treatment with cycloheximide. Mannose rich N-glycosylated synaptophysin was detected by treating retinal lysates with endoglycosidase H followed by immunoblot analysis. Synaptophysin mRNA translation was significantly increased after 1 month (*p*<0.001) and 2 months (*p*<0.05) of STZ-diabetes, compared to age-matched controls. Newly synthesized synaptophysin degradation was significantly accelerated in the retina after 1 and 2 months of diabetes compared to controls (*p*<0.05). Mannose rich glycosylated synaptophysin was significantly increased after 1 month of STZ-diabetes compared to controls (*p*<0.05).These data suggest that diabetes increases mRNA translation of synaptophysin in the retina, resulting in an accumulation of mannose rich glycosylated synaptophysin, a transient post-translational state of the protein. This diabetes-induced irregularity in post-translational processing could explain the accelerated degradation of retinal synaptophysin in diabetes.

## Introduction

Synaptophysin is an abundant transmembrane protein in presynaptic neurotransmitter vesicles of neurons [1], [2]. It is a glycoprotein with four transmembrane domains and both amino and carboxyl termini face the cytoplasm [3], [4], [5]. Although the function of synaptophysin is not fully understood, it is known to interact with other synaptic proteins including the v-SNARE vesicle-associated membrane protein 2/synaptobrevin II (VAMP2), suggesting a role in vesicle docking and neurotransmitter release [6], [7], [8]. Synaptophysin has also been implicated in the recycling of synaptic vesicles by associating with dynamin I, a GTPase required for endocytosis [9].

During its post-translational processing, synaptophysin is N-glycosylated in the endoplasmic reticulum (ER) [10]. Soon after mRNA translation, a mannose rich oligosaccharide is added to the new peptide chain [11]. Cell culture studies suggest that this mannose rich synaptophysin is a transient state of the protein which is demannosylated during several steps in the ER and Golgi apparatus [12]. In the Golgi, the removal of 2 mannose residues by Golgi alpha-mannosidase II is the final step in the transformation of synaptophysin from its mannose rich form into a highly complex N-glycan [12], [13]. The complex N-glycosylated synaptophysin is then released from the *trans* Golgi.

Synaptophysin is abundant in the retina. In Sprague-Dawley rats and humans, synaptophysin immunoreactivity is distributed throughout the synapse-rich inner and outer plexiform layers of the retina [14], [15], [16]. In rod photoreceptors of synaptophysin knockout mice, synaptic vesicles are reduced in number and have increased diameters [17]. Given the absence of the compensatory isoform synaptoporin, in mouse photoreceptors, these data suggest that synaptophysin has an important role in synaptic vesicle recycling and formation in the rodent retina [18], [19], [20], [21], [22].

Studies in rodent models suggest that diabetes causes neurodegeneration in the retina. In situ DNA terminal dUTP nick end labeling (TUNEL) identified a 10-fold increase in the frequency of apoptosis in whole-mounted rat retinas after 1, 3, 6, and 12 months of diabetes [23]. In this study most apoptotic cells were not associated with blood vessels, suggesting that retinal neural cell death is increased by diabetes. After 7.5 months of hyperglycemia, the thickness of the inner plexiform layer was reduced by 22% of control and the inner nuclear layer was reduced by 14% of control [23]. A significant decrease in the thickness of the whole retina and the inner and outer nuclear layers was also seen in 10-week streptozotocin (STZ)-diabetic mice [24]. In retinas from rats after 2, 8, and 16 weeks of STZ-diabetes, 2% to 6% of caspase-3 immunoreactive cells were double-labeled for tyrosine hydroxylase immunoreactivity. The same study also reported a 20% decrease in the number of cholinergic and a 16% decrease in dopaminergic amacrine cells in retinas from Ins2^Akita^ diabetic mice, compared with the non-diabetic controls [25]. Collectively, increased caspase activity and neural cell loss observed in these studies indicate retinal neurodegeneration due to diabetes.

Another indication of neurodegeneration may be a change in neuron-specific protein expression. A recent study reported that 1 month of STZ-diabetes decreased the size and density of synaptophysin-immunoreactive puncta in both retinal plexiform layers. Immunoblot analysis indicated that after 1 month of STZ-diabetes several presynaptic proteins, including synaptophysin, were significantly reduced in the rat retina compared to age-matched controls [14]. Loss of synaptophysin protein immunoreactivity and content may indicate decreased vesicle recycling, given the role of synaptophysin in vesicle endocytosis [9], [26].

In this study we examine mechanisms responsible for the reduced expression of retinal synaptophysin in diabetic rats. mRNA translation, degradation, and glycosylation of synaptophysin are measured in retinas from diabetic Sprague-Dawley rats using an explant system. The results suggest that diabetes increases the mRNA translation of synaptophysin while its post-translational glycosylation is increased, and its degradation is accelerated.

## Methods

### Ethics Statement

All animal experiments were approved by the Penn State College of Medicine Institutional Animal Care and Use Committee and performed in accordance with the NIH Guidelines for the Care and Use of Laboratory Animals and the Association for Research in Vision and Ophthalmology Statement for the Use of Animals in Ophthalmic and Vision Research.

### Materials

Streptozotocin (STZ), sodium citrate, and general buffers and chemicals were purchased from Sigma Aldrich (St Louis, MO, USA). Complete™ protease inhibitor tablets were purchased from Roche Diagnostics (Mannheim, Germany).

### Animals

Male Sprague-Dawley rats, 150–175 g (Charles River Laboratories, Wilmington, MA, USA), were housed in the Penn State Hershey College of Medicine Animal Facility. At a weight of 270–300 g, diabetes was induced by single intraperitoneal STZ injection (65 mg/kg body weight in 10 mM sodium citrate, pH 4.5). Control rats received an equal volume of citrate buffer. Diabetes was confirmed 6 days later by a blood glucose level >250 mg/dL in a drop of blood from the tail (Alphatrak, Abott Laboratories, Illinois, USA. This meter is calibrated specifically for rat blood). Rats were housed on a 12 h light/dark schedule with *ad libitum* food and water. Immediately prior to sacrifice rats were weighed and their blood glucose levels were recorded. Animals were killed by anesthesia (ketamine 66.7 mg/kg/xylazine 6.7 mg/kg, intramuscular) and decapitation. There were no fatalities in either control or diabetic groups prior to sacrifice.

### Immunoblot Analysis

Retinas were sonicated in lysis buffer (100 mM NaCl, 1.0% Triton X-100, 0.5% sodium deoxycholate, 0.2% sodium dodecylsulfate, 2 mM EDTA, 10 mM HEPES, 1 mM sodium orthovanadate, 10 mM sodium fluoride, 10 mM sodium pyrophosphate, 1 mM benzamidine, 10 µM microcystin, and one Complete™ protease inhibitor tablet/10 ml of buffer, pH 7.3) and then rocked for 15 min at 4°C. Samples were centrifuged (12 mins at 12,500 g, 4°C) and supernatant protein concentrations were determined using the BCA protein assay (Bio-Rad Laboratories, Hercules, CA, USA). Equal amounts of protein were adjusted to equal volumes diluted in sample buffer (NuPAGE® LDS, Invitrogen, Carlsbad, CA, USA) and resolved on 4–12% Bis-Tris gels (NuPAGE® Novex). Protein bands were transferred to nitrocellulose membranes (Thermo Fisher, Waltham, MA, USA), blocked with 5% milk in Tris-buffered saline with 0.05% Tween-20, and incubated with primary antibody overnight at 4°C. Blots were incubated with appropriate secondary antibodies for 1 hr at room temperature. Blots were imaged with a Typhoon 8600 Variable Mode Imager (GE Healthcare®, Wisconsin, USA), and quantified using Image Quant software (Molecular Dynamics, California, USA), standardized to β-actin, and expressed as percentage of control ± SEM.

### Antibodies

The rabbit polyclonal antibodies against eukaryotic initiation factors (eIFs) 2α, 2Bε, 3A, and 4G were purchased from Cell Signaling Technology (Danvers, Massachusetts, USA). The mouse monoclonal anti-synaptophysin (SVP-38) was purchased from Sigma Aldrich (St Louis, MO, USA). Anti-rabbit and anti-mouse alkaline phosphatase-conjugated secondary antibodies were used (Amersham Pharmacia Biotech, Piscataway, NJ, USA).

### ^35^S-met/cys Metabolic Labeling and Degradation Studies

Retinas were rapidly extracted and incubated in 1 ml of methionine-free DMEM (Sigma Aldrich) in a 12 well plate for 20 min in a cell culture incubator (37°C). After the pre-incubation period, 100 µCi of ^35^S-methionine/cystine protein labeling mix (EasyTag™ EXPRESS, Perkin Elmer, Waltham, Massachusetts, USA) was added to the medium and incubated for 30 min. For the pulse-chase experiments, the retinas were then washed and incubated in DMEM containing 1 mM methionine (Sigma Aldrich) for 0, 30, 60, 90, or 120 min. To measure the loss of total synaptophysin in the retina, explants were incubated in DMEM containing methionine and 30 mM cycloheximide (CHX) (Sigma Aldrich) for 30, 60, or 120 min.

### Immunoprecipitation and Autoradiography

Retinas were homogenized in IP buffer (50 mM HEPES, 137.5 mM NaCl, 1 mM MgCl_2_, 1 mM CaCl_2_, 10 mM Na_2_H_2_P_2_O_7_, 10 mM EDTA, 10 mM NaF, 2 mM PMSF, 0.16% benzamidine, 10% glycerol, 1% NP-40, and one Complete™ protease inhibitor tablet for every 10 ml of buffer) and centrifuged at 12,500 g at 4°C for 10 min. The protein concentration was determined and synaptophysin was immunoprecipitated using 450 µg of lysate and 20 µg of rabbit polyclonal anti-synaptophysin (Novus Biologicals, Littleton, Colorado, USA), rotated overnight at 4°C. The following day, 150 µl of protein A sepharose beads (Sigma Aldrich) were added to the lysate/antibody mix and rotated for 3 hr at room temperature. The beads were washed three times with IP buffer, resuspended in 40 µl of 1X NuPAGE® LDS sample buffer (Invitrogen®, California, USA), and boiled for 5 min. Samples were centrifuged and the supernatants were run on 10% Bis-Tris gels (NuPAGE® Novex). The gel was fixed (30% methanol, 10% glycerol) for 1 hr and dried overnight at 55°C. Dried gels were placed in phospho-screens for 72 hr and quantified as described above. For measurement of total mRNA translation, whole retina lysates were run on the gel followed by autoradiography.

### Perchloric Acid (PCA) Precipitation

For PCA precipitation, 250 µl of retinal lysate was added to 1.25 ml of 1N PCA and the sample was boiled for 15 min. The resulting precipitate was washed twice by centrifugation (10 min at 3,200 g, 4°C) followed by a wash with 1.5 ml of 1∶2∶1 chloroform: ethanol: ether and lastly with 1.5 ml of ether only. Samples were allowed to dry overnight before being resuspended in 1.5 ml of 0.1 N NaOH and boiled for 10 min. 500 µl of sample and 5 ml of scintillation fluid (Formula 989, Perkin Elmer, Waltham, Massachusetts, USA) were mixed in scintillation vials and counted (LS 6500 Beckman Coulter, California, USA). Data were expressed as CPM (counts per min)/µg protein/hr.

### Endo H Treatment of Retinal Lysates

6 µl of 1X denaturing buffer (New England Biolabs, Ipswich, MA, USA) was added to 30 µg of retinal lysate and boiled for 7 min. 15 µg was taken from each sample and incubated with endoglycosidase H (endo H) and 1X ‘G5’ reaction buffer (New England Biolabs, Ipswich, MA, USA) for 1 hr at 37°C. The reaction was stopped by the addition of sample buffer (1X NuPAGE® LDS, Invitrogen®, California, USA) and heated at 70°C for 15 min. The samples were probed for synaptophysin through immunoblot analysis.

### Statistical Analysis

Data are expressed as mean ± SEM. Statistical comparisons between control and diabetic groups were made by Student’s two-tailed homoscedastic t-test (Microsoft Excel; Microsoft, Redmond, WA, USA). Two-way ANOVA followed by Bonferroni multiple comparisons test was used for the newly synthesized and total synaptophysin depletion assays (Prism; Graphpad, La Jolla, CA, USA).

## Results

### Animals

The terminal weight, % weight gain, and blood glucose levels in control (CNT) and diabetic (STZ) rats are reported in Table 1. The STZ diabetic rats gained significantly less weight than the controls (p<0.001), although the measure of % weight gain was always positive, indicating no weight loss in any group of rats. The STZ-diabetic rats also had significantly higher blood glucose levels compared to age-matched controls (*p*<0.001).

**Table 1:** Terminal weight and blood glucose of diabetic and control animals.

	1 month			2 months		
Group	Weight (g)	Weight Gain (%)	Blood Glucose (mg/dl)	Weight (g)	Weight Gain (%)	Blood Glucose (mg/dl)
**CNT**	523.7±10	79	155.0±3	564.0±9	102	140.8±2
**STZ**	326.5±8***	15	683.8±15***	334.4±9***	18	615.2±20***

The % weight gain  =  ((terminal weight – weight at diabetes induction)/weight at diabetes induction). Student’s two-tailed t-test was used to determine significance. CNT  =  control, STZ  =  diabetic.

*** *p*<0.001 compared to CNT.

### Experiment 1. ^35^S-met/cys Protein Incorporation is Increased in Retinal Explants from 1-Month STZ-diabetic Rats Compared to Controls

**Figure 1:**
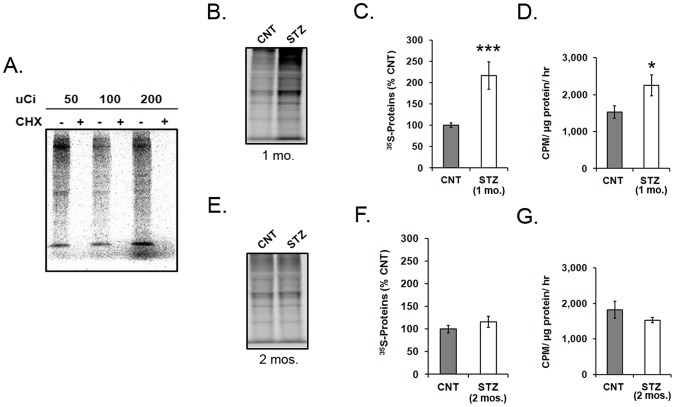
mRNA translation in explant retinas form diabetic and control rats. Explant retinas were incubated with ^35^S-met/cys for 30 min to label newly translated proteins. A) Explants were incubated in 50, 100, and 200 µCi of ^35^S-met/cys. Addition of 30 mM cycloheximide (CHX) to the medium abolished the mRNA translation of total protein. Autoradiography and PCA precipitation were performed on explants from 1 and 2-month diabetic and control rats following ^35^S-met/cys incubation. B) ^35^S-met/cys incorporated into many proteins in 1-month diabetic and age-matched control explants. C) ^35^S-met/cys incorporation was increased in retinal explants from 1-month STZ-diabetic rats (*n* = 15) compared to age-matched controls (*n* = 13) (***p<0.001). D) The average CPM in explants from 1-month STZ-diabetic rats was significantly increased compared to age-matched controls (*n* = 9; *p<0.05). E) ^35^S-met/cys incorporation in explants from 2-month diabetic and age-matched control rats. F) ^35^S-met/cys incorporation was not significantly different in retinal explants from 2-month STZ-diabetic rats compared to age-matched controls (*n* = 9). G) The average CPM was not significantly different in explants from 2-month STZ-diabetic rats compared to age-matched controls (*n* = 9).

An explant radioactive metabolic labeling assay was used to measure mRNA translation in the rat retina. Amino acid incorporation was completely inhibited by cycloheximide when retinas were incubated with 50, 100, and 200 µCi ^35^S-Met/Cys, confirming that the radioactive incorporation was due to mRNA translation and not amino acid transport/storage (Fig. 1A).

mRNA translation was measured in retinas from 1-month STZ-diabetic rats and age-matched controls followed by autoradiography of lysates (Fig. 1B). ^35^S-met/cys incorporation was significantly elevated in retinas from 1-month STZ-diabetic rats compared to controls (2.31 fold; *p*<0.001; Fig. 1C). ^35^S-met/cys incorporation was also measured by PCA precipitation. The average CPM in samples from 1-month STZ-diabetic rats was significantly more than age-matched controls (2,255 CPM/µg protein/hr vs. 1,531 CPM/µg protein/hr) (*p*<0.05; Fig. 1D). ^35^S-met/cys incorporation was also measured in retinas from 2-month STZ-diabetic and age-matched control rats (Fig. 1E). Incorporation was not significantly different in retinas from 2-month STZ rats compared to controls (Fig. 1F). The average CPM was also not significantly different (1,530CPM/µg protein/hr in 2-month STZ-diabetic rats compared to 1,827 CPM/µg protein/hr in controls) (Fig. 1G).

### Experiment 2. Protein Content of eIFs is not Changed, but 4EBP-1 Phosphorylation is Lower in STZ-diabetic Rat Retinas Compared to Controls

**Figure 2:**
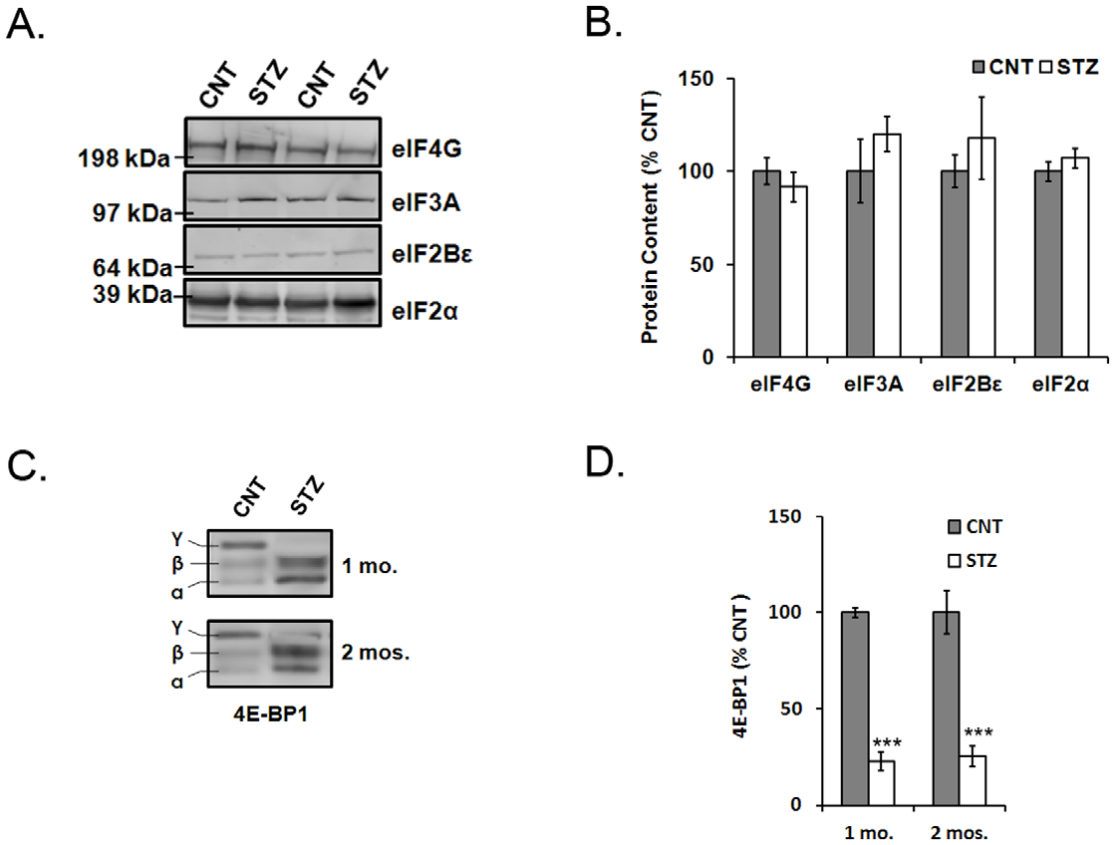
Expression of eIFs in retinas of diabetic and control rats. eIF2α, eIF2Bε, eIF3A, and eIF4G were quantified in whole retina lysates from 1-month STZ-diabetic and age-matched control rats. Hyperphosphorylated 4E-BP1 content was also measured in 1- and 2-month STZ-diabetic and age-matched control rat retinas. A) eIF2α resolved as a single 38 kDa band, eIF2Bε as a single 85 kDa band, eIF3A as a single 166 kDa band, and eIF4G as a single 220 kDa band. B) The content of each of the eIFs was not significantly different in the whole retina from 1-month of STZ diabetic rats compared to controls (*n* = 10). C) 4EBP-1 resolved as 3 bands between 15 and 20 kDa. The sum of the α and β bands was divided by the γ band to quantify phosphorylation. D) Phosphorylated 4EBP-1 was significantly reduced in the rat retina after 1 month and 2 months of STZ-diabetes compared to controls (*n* = 10; ***p<0.001).

eIF2α, eIF2Bε, eIF3A, and eIF4G proteins were quantified in retinal lysates from 1-month STZ-diabetic and age-matched control rats (Fig. 2). eIF2α resolved as a single 38 kDa band, eIF2Bε as a single 85 kDa band, eIF3A as a single 166 kDa band, and eIF4G as a single 220 kDa band (Fig. 2A). In all cases, the content of these proteins was not significantly different in retinas from 1-month STZ-diabetic rats compared to controls (Fig. 2B). 4EBP-1 phosphorylation was measured in retinas from 1- and 2-month STZ-diabetic and age-matched control rats. 4EBP-1 resolved as 3 bands (α, β, and γ) between 15 and 20 kDa (Fig. 2C). After 1 and 2 months of STZ-diabetes the amount of phosphorylated 4EBP-1 in the rat retina was significantly less than controls (*p*<0.001; Fig. 2D).

### Experiment 3. ^35^S-met/cys Incorporation into Retinal Synaptophysin is Greater in STZ-Diabetic Rats Compared to Controls

**Figure 3:**
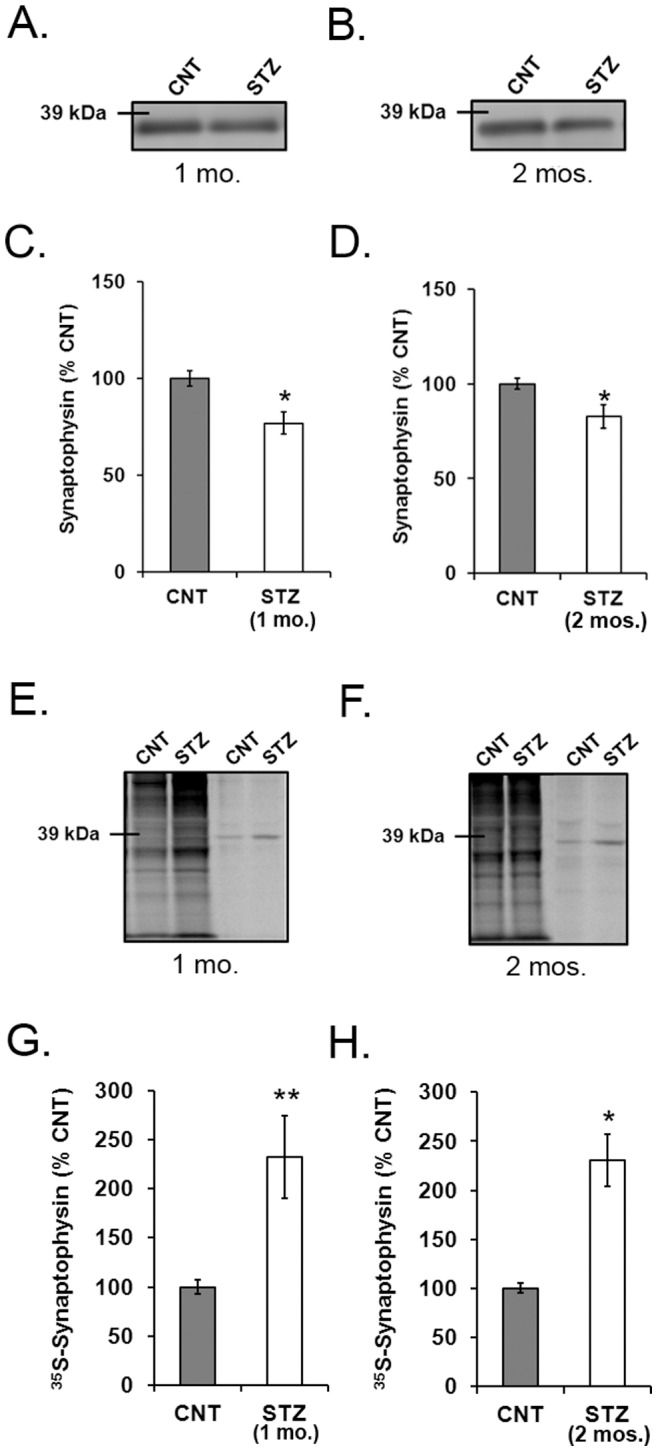
Expression and mRNA translation of synaptophysin in retinas of diabetic and control rats. Relative total and ^35^S-synaptophysin content was measured in retina lysates by immunoblot or autoradiogram after immunoprecipitation. A) Retinal synaptophysin resolved as a single 38 kDa band in lysates from 1-month STZ-diabetic rats and age-matched controls. B) Retinal synaptophysin also resolved as a single 38 kDa band in lysates from 2-month STZ-diabetic rats and age-matched controls. C) Retinal synaptophysin was significantly less in 1-month STZ-diabetic rats (*n* = 15) compared to age-matched controls (*n* = 13) (*p<0.05). D) Retinal synaptophysin content was also significantly less in 2-month STZ-diabetic rats compared to age-matched controls (*n* = 9; *p<0.05). E)^ 35^S-met/cys synaptophysin bands were detected at 38 kDa in retinas from 1-month STZ and control rats. F) ^35^S-met/cys synaptophysin bands were also detected at 38 kDa in retinas from 2-month STZ and control rats. G) ^35^S-met/cys synaptophysin significantly increased in retinas from 1-month STZ-diabetic rats (*n* = 15) compared to age-matched controls (*n* = 13) (**p<0.01). H) ^35^S-met/cys synaptophysin was significantly increased in retinas from 2-month STZ-diabetic rats compared to age-matched controls (*n* = 9; *p<0.05).

Synaptophysin protein content was measured by immunoblot analysis in retinal lysates from 1- and 2-month STZ-diabetic and age-matched control rats (Fig. 3A, B). There was significantly less synaptophysin in the 1- and 2-month STZ-diabetic rat retinas compared to controls (*p*<0.05; Fig. 3C, D), confirming previously published results [14].

Synaptophysin mRNA translation in the retinal explants was determined by 100 µCi ^35^S-Met/Cys incorporation for 30 min, followed by synaptophysin immunoprecipitation and autoradiography (Fig. 3E, F). ^35^S-met/cys synaptophysin in 1-month STZ diabetic rat retinas was significantly greater than controls (2.3 fold; *p*<0.01; Fig. 3G). ^35^S-met/cys incorporation into synaptophysin in 2-month STZ-diabetic rat retinas was also significantly greater than controls (2.3 fold; *p*<0.05; Fig. 3H).

### Experiment 4. Newly Synthesized Synaptophysin is Depleted Faster in Retinal Explants from STZ-diabetic Rats Compared to Controls

**Figure 4:**
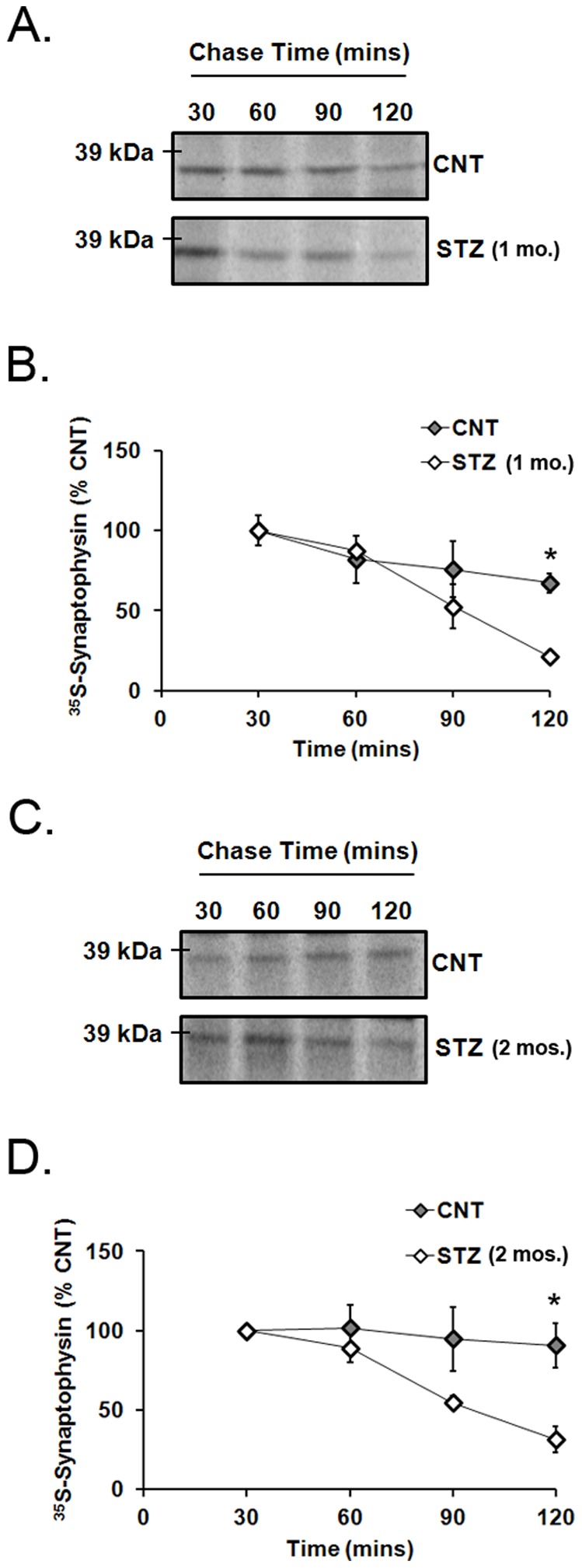
Degradation of newly synthesized ^35^S-synaptophysin in explant retinas from diabetic and control rats. Retinas were incubated with ^35^S-met/cys for 30 min, followed by 1 mM cold methionine for 30, 60, 90, or 120 min followed by autoradiography. A) ^35^S-met/cys synaptophysin resolved as a single 38 kDa band in retinas from 1-month STZ-diabetic and age-matched control rats. B) ^35^S-met/cys synaptophysin was significantly less in retinas from 1-month STZ-diabetic rats compared to controls after 120 min (*n* = 4; *p<0.05). C) ^35^S-met/cys synaptophysin resolved as a single 38 kDa band in retinas from 2-month STZ-diabetic and age-matched control rats. D) ^35^S-met/cys synaptophysin was also significantly less in retinas from 2-month STZ-diabetic rats compared to controls after 120 min (*n* = 4; *p<0.05).

The loss of newly synthesized synaptophysin in whole explant retinas from 1-month STZ-diabetic and age-matched control rats was measured using a pulse-chase approach. After the incubation of retinal explants in ^35^S-met/cys for 30 min, which was then replaced by non-radioactive methionine for 0, 30, 60, 90, or 120 min, ^35^S-met/cys-synaptophysin was quantified by immunoprecipitation and autoradiography. There was significantly less ^35^S-met/cys synaptophysin in retinas from 1-month of STZ-diabetic rats after 120 min compared to age-matched controls (p<0.05; Fig. 4A, B). A similar result was also found in the 2-month STZ-diabetic rats compared to age-matched controls (p<0.05; Fig. 4C, D).

### Experiment 5. Retinal Synaptophysin is Depleted Faster in 2-month STZ-diabetic Rats Compared to Controls

**Figure 5:**
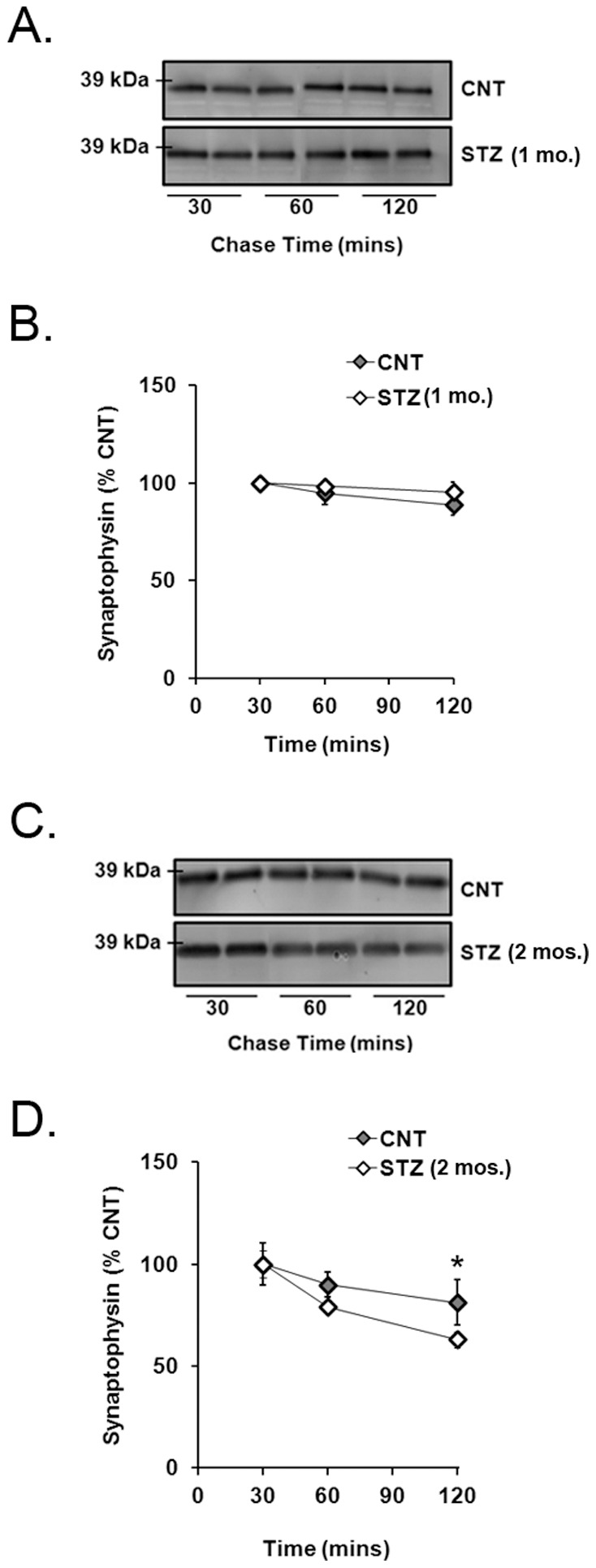
Degradation of synaptophysin in explant retinas from diabetic and control rats. Synaptophysin content was measured by immunoblot analysis on retinas incubated with cycloheximide for 30, 60, or 120 min. A) Synaptophysin resolved as a 38 kDa band in retinas from 1-month STZ-diabetic rats and age-matched controls after 30, 60, and 120 min. B) There was no significant difference in synaptophysin content between retinas from 1-month STZ-diabetic rats and controls after 120 min (*n* = 6). C) Synaptophysin resolved as a 38 kDa band in retinas from 2-month STZ-diabetic rats and age-matched controls after 30, 60, and 120 min. D) There was significantly less synaptophysin in retinas from 2-month STZ-diabetic rats compared to controls after 120 min (*n* = 6; *p<0.05).

Retinas from 1- and 2-month STZ-diabetic and age-matched control rats were treated with cycloheximide for 30, 60, or 120 min, and synaptophysin was quantified by immunoblot analysis. The amount of synaptophysin in retinas from 1-month STZ-diabetic rats was not significantly different compared to age-matched controls after 120 min (Fig. 5 A, B). The amount of synaptophysin was significantly less in retinas from 2-month STZ-diabetic rats compared to controls after 120 min of protein synthesis inhibition (p<0.05; Fig. 5 C, D).

### Experiment 6. Mannose Rich N-glycosylated Synaptophysin is Increased in Retinas from 1-month STZ-diabetic Rats Compared to Controls

**Figure 6:**
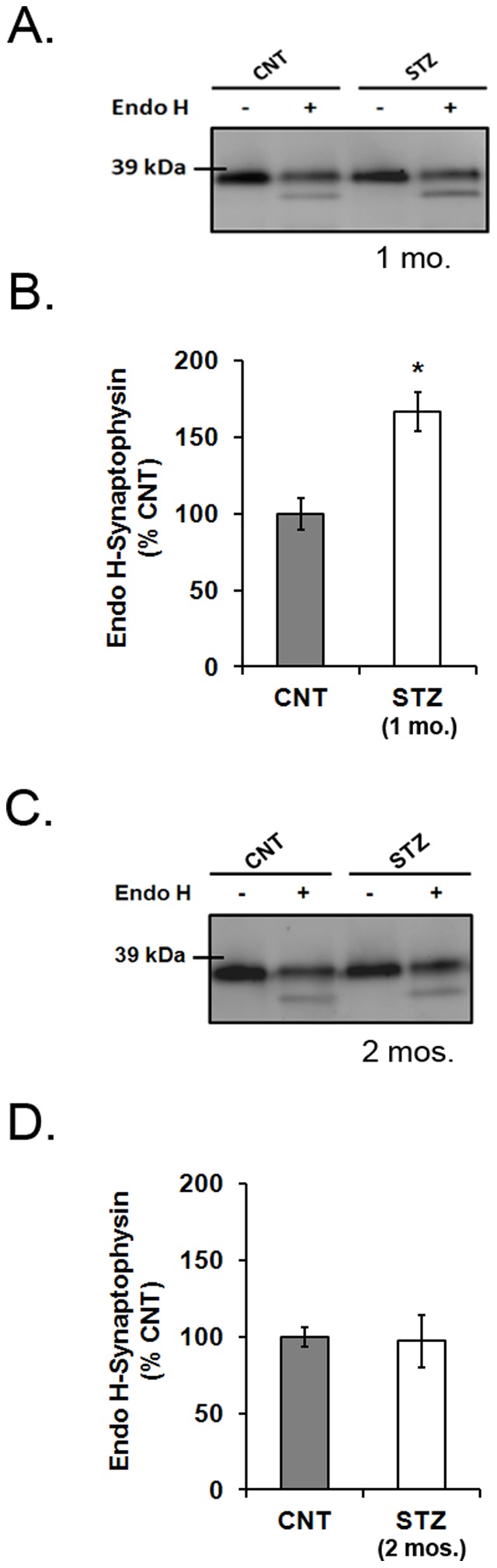
The content of mannose rich (endo H sensitive) synaptophysin in retinas from diabetic and control rats. Retinal lysates were treated with endo H to quantify mannose rich glycosylated synaptophysin. A) Endo H treated samples from 1-month STZ-diabetic rats and controls had an additional band at 35 kDa below the 38 kDa synaptophysin band. B) There was a significantly more endo H-sensitive synaptophysin in the retinal lysates from 1-month STZ-diabetic rats compared to age-matched controls (*n* = 8; *p<0.05). C) Endo H treated samples from 2-month STZ-diabetic rats and controls also had an additional band at 35 kDa below the 38 kDa synaptophysin band. D) There was no significant difference in endo H-sensitive synaptophysin between retinal lysates from 2-month STZ-diabetic rats compared to age-matched controls (*n* = 8).

Endo H was used to cleave mannose rich oligosaccharides from synaptophysin. Retinal lysates from 1- and 2-month STZ-diabetic and age-matched control rats were treated with endo H and immunoblotted for synaptophysin. There was significantly more endo H-sensitive synaptophysin in the retinas from 1-month STZ-diabetic rats compared to age-matched controls (*p*<0.05; Fig. 6 A, B). There was no significant difference in the amount of endo H-sensitive synaptophysin in the retinas from 2-month STZ-diabetic rats compared to age-matched controls (Fig. 6 C, D).

## Discussion

Previous data showed that STZ-diabetes significantly reduced the content and immunoreactivity of inner and outer plexiform layer synaptophysin, and several other synaptic proteins in the rat retina [14]. To cause this reduction, diabetes may target molecular mechanisms responsible for synaptophysin regulation. Since the relative mRNA content of synaptophysin was unchanged after 1 month of diabetes [14], the mRNA translation, protein glycosylation and degradation in the retina were identified as potential molecular mechanisms for the reduction. It was hypothesized that STZ-diabetes alters synaptophysin mRNA translation and N-glycosylation in the rat retina. An explant radiolabeling assay was developed to test mRNA translation and synaptophysin degradation. N-glycosylation was measured using endo H treatment and immunoblot analysis.

In the autoradiography study, the total mRNA translation was significantly increased in retinal explants after 1 month of diabetes. After 2 months of diabetes, however, the total mRNA translation was not different from controls. In addition to autoradiography, these results were replicated using PCA precipitation. While results of previous studies imply that diabetes reduces mRNA translation in the retina [27], it is important to note that the radioactive explant assay in this study will likely only label a subset of proteins that are rapidly synthesized to detectable levels within a 30 minute time frame. Therefore, the results of this study suggest that 1-month of diabetes increases the expression of rapidly synthesized proteins in the rat retina. In order to explain the increase in total mRNA translation in the 1-month group, eIFs involved in the regulation of translation were examined by immunoblot analysis. The content of eIF2α, eIF2Bε, eIF3A, and eIF4G was not different between retinas from STZ-diabetic rats and age-matched controls. Similarly, a previous study reported that the phosphorylation state of eIF2α and eIF4G is unaltered by diabetes [27]. Therefore, content and phosphorylation of these eIFs may not be responsible for the altered retinal mRNA translation seen in this study. There was however, a significant decrease in the amount of phosphorylated 4E-BP1 in the rat retina after 1- and 2- months of diabetes compared to controls. In the hypophosphorylated state, 4E-BPs compete with eIF4G for binding to eIF4E and prevent eIF4F complex assembly leading to a decrease in cap-dependent mRNA translation [28], [29]. In the current study, mRNA translation was increased after 1 month of diabetes, suggesting that the retinal mRNA translation detected in this assay is likely not regulated by 4E-BP1.

Other studies have found increased mRNA translation of select proteins in response to elevated glucose. In prediabetic BB rat pancreas, nitric oxide synthase mRNA translation is increased in areas of inflammation [30]. Cell culture studies on renal epithelial cells indicate that synthesis of laminin-β1, a glycoprotein that contributes to renal extracellular matrix expansion in diabetes, is increased within minutes of exposure to high glucose [31]. In the present study on rat retinas, STZ-diabetes significantly increased synaptophysin mRNA translation compared to age-matched controls. Evidently, while total mRNA translation in the retina eventually stabilizes after the induction of diabetes, synaptophysin mRNA translation continues at a higher rate. While the reason for increased synaptophysin mRNA translation is not known, it could be speculated that increased mRNA translation is a compensatory mechanism for the reduction of the mature protein.

The total amount of synaptophysin was reduced by diabetes, as shown previously (14), while the mRNA translation of newly synthesized ^35^S-synaptophysin was elevated (Figure 3). These data introduce a dilemma that can only be explained by an increase in synaptophysin degradation. Diabetes significantly depleted the newly synthesized ^35^S-synaptophysin compared to controls (Fig. 4), offering a potential explanation. Degradation of total synaptophysin in 2-month diabetic rats was also accelerated, but did not become significant in retinas from 1-month diabetic rats, at least during the short 2 hour time course used for this study (Fig. 5), suggesting that the rate of degradation of ^35^S-synaptophysin increases with extended duration of diabetes. These data suggest that the rate of mRNA translation for synaptophysin is elevated within the first month of diabetes, but that there is also an increase in its degradation during an early stage of its maturation, possibly during its post-translational processing.

It can be speculated that increased degradation of newly synthesized ^35^S-synaptophysin is a result of irregular processing of the glycoprotein in the ER and/or Golgi apparatus, because failure of glycoproteins to fold correctly during post-translational processing can trigger early degradation [32]. Folding and proper processing of a glycoprotein is dependent on its glycosylation soon after mRNA translation. Increases in synaptophysin mRNA translation in the retina after diabetes prompted an investigation into synaptophysin glycosylation. Newly synthesized synaptophysin is N-glycosylated in the lumen of the ER with a 14 sugar molecule consisting of 2 N-Acetylglucosamine (GlcNAc), 9 mannose, and 3 glucose residues [11]. The mannose rich synaptophysin, which is sensitive to deglycosylation by endo H, is then exported and processed through several compartments of the Golgi apparatus. In the *trans* Golgi, the removal of 2 mannose residues by Golgi alpha-mannosidase II is the final step in the transformation of synaptophysin from its ‘immature” form into a highly complex N-glycan [12]. The “mature” synaptophysin then becomes insensitive to deglycosylation by endo H. This mature form is released from the *trans* Golgi. In this study, we report that “immature” glycosylated synaptophysin was significantly increased in rat retinas after 1 month of STZ-diabetes compared to age-matched controls. These data suggest that the accumulation of glycosylated synaptophysin may result in improper post-translational processing of the protein. This in turn may increase activity of synaptophysin degradation mechanisms. Further, ER stress can result from the accumulation of unfolded or misfolded proteins. A previous study has reported that diabetes increases the activity of inositol-requiring enzyme 1α (IRE1α) and PKR like ER kinase (PERK), two major transmembrane transducers sensing ER stress, in retinas of Ins2^Akita^ mice compared to wild-type litter mates [33]. Therefore, ER stress is one potential explanation for the early degradation of retinal synaptophysin in 1-month STZ diabetic rats.

Post-translational processing of synaptophysin requires several transient glycosylation steps. Incubation with the enzyme, endo H, identified the transient mannose rich form of synaptophysin. The increase in endo H sensitive synaptophysin after 1-month of diabetes could explain the accelerated degradation of ^35^S-synaptophysin at this time point; however the lack of change in endo H-sensitive synaptophysin in the retinas of 2-month diabetic rats does not. We offer the possibility that early posttranslational degradation occurs in 1-month animals as a result of excessive mannosylation, while the degradation and reduced content of synaptophysin after 2-months may be triggered by a different mechanism.

Together, these data suggest that the previously reported reduction in synaptophysin in the rat retina due to diabetes is attributable to dysregulation of post-translational processing. Diabetes likely increases mannose rich glycosylated synaptophysin content in the retina. Increased presence of this “immature” form of the protein implies an irregularity in post-translational processing. In conclusion, the data presented in this study suggest that synaptophysin turnover and post-translational processing in the rat retina is compromised by experimental diabetes. The reduction of synaptophysin is likely due to overcompensation by its degradation mechanism in response to the accumulation of “immature” glycosylated synaptophysin content.
